# Shear Wave MR Elastography of the Orbit: Preliminary Findings

**DOI:** 10.3390/diagnostics15182342

**Published:** 2025-09-16

**Authors:** Ayden L. Olsen, Daniel T. Ginat

**Affiliations:** 1 Pritzker School of Medicine, The University of Chicago, Chicago, IL 60637, USA; ayden.olsen@uchicagomedicine.org; 2 Department of Radiology, Section of Neuroradiology, The University of Chicago, Chicago, IL 60637, USA

**Keywords:** MR elastography, shear wave, orbit, technique

## Abstract

Magnetic resonance elastography (MRE) can provide insight into the biomechanical properties of tissues. Yet, there is a lack of widespread utilization of MRE for evaluation of the head and neck region, particularly the orbit. This article describes the preliminary MRE findings for imaging of the orbit in healthy human subjects and evaluates its feasibility and technical considerations. Two healthy volunteers were recruited for participation. A standard liver driver was positioned over the forehead of the volunteer, after which a 3T scanner was used to obtain MRE images of the orbit at 8 and 20 kPa. Resulting image quality and strain patterns were assessed. Basic viscoelastic information was visualized on orbit elastograms obtained at both 8 and 20 kPa, with both image sets displaying increased strain in the posterior globes. Image quality appeared better at 8 kPa than at 20 kPa. While the development of specialized devices and techniques requires further investigation to optimize image quality, MRE is feasible for effectively visualizing viscoelastic properties of intra- and periorbital tissues.

**Figure 1:**
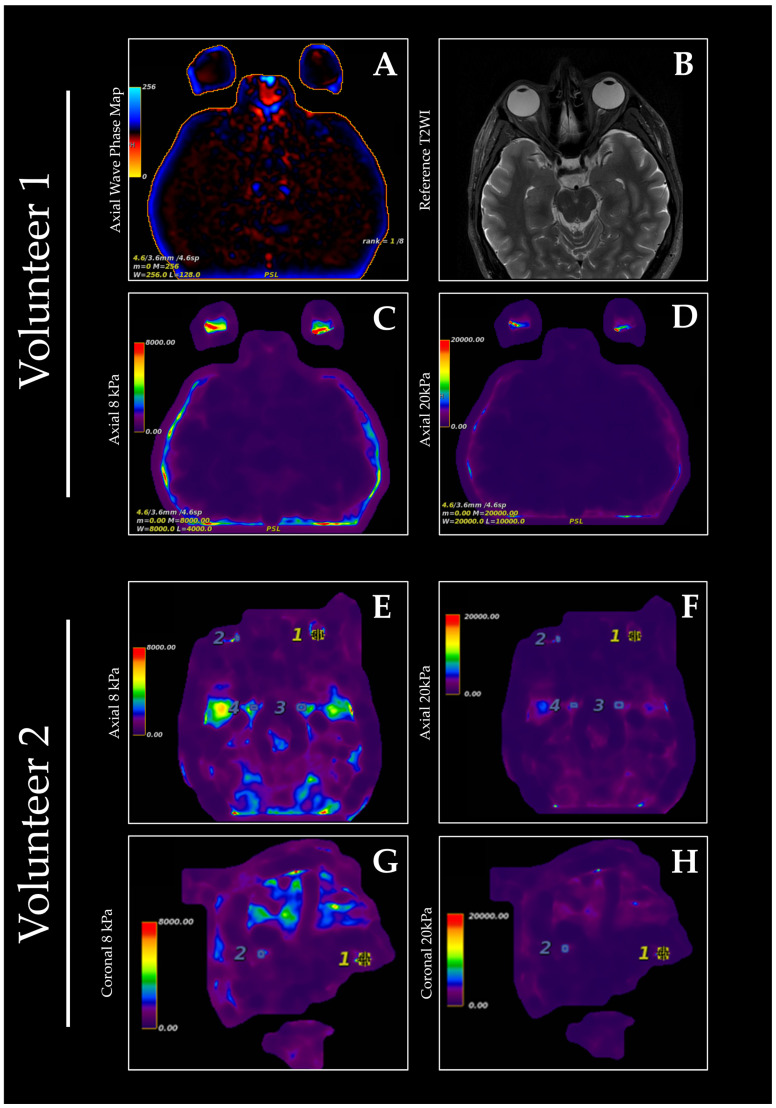
Orbital MRE was performed on two healthy volunteers, who reported minimal discomfort during the scan. Scanning was performed utilizing a 3T MRI scanner (GE Signa HDx; GE Healthcare, Milwaukee, WI, USA), a neurovascular coil, and a liver driver. Immediately prior to scanning, the driver was positioned on the forehead of the subject. A GEM flex coil was then positioned on top of the liver driver. MRE data were acquired with a 2D axial gradient-recalled echo (GRE) sequence with a field of view of 300 mm × 300 mm at 8 and 20 kPa. The driver amplitude was set to 60 Hz. Axial wave phase mapping (**A**) shows the raw phase information used in subsequent parallel imaging reconstruction. Reference axial T2-weighted images were also acquired during the same scanning session (**B**). Qualitatively, image quality appeared to be better at 8 kPa versus 20 kPa and demonstrated greater sensitivity to subtle variations in stiffness within the orbital tissues (**C**,**E**,**G**); however, the 20 kPa setting facilitated the characterization of the viscoelastic properties of stiffer tissues within the globe (**D**,**F**,**H**). Overlay maps were then created utilizing the elastogram data and the reference T2 and LAVA sequence scans for volunteers one and two, respectively, which localize the areas of highest strain within the orbit to the posterior globe. MRE is a noninvasive imaging technique that measures tissue stiffness by analyzing the propagation of mechanical vibration with motion-sensitive gradients [1]. In particular, the viscoelastic properties of tissues, such as the shear modulus, can be determined by analyzing their wave-propagation characteristics [2]. An actuating mechanism is used to generate the necessary tissue displacement, but the magnetic resonance imaging (MRI) environment imposes certain constraints on the design of such devices [3]. MRE has previously been applied to various structures of the head and neck, including the thyroid gland, parotid gland, lymph nodes, tongue, and palate [4,5,6,7]. Multiple studies have shown the ability of MRE to differentiate between normal and diseased thyroid tissue, including Hashimoto’s thyroiditis and thyroid cancer [4,5]. Other studies have used MRE to determine viscoelastic properties of the brain [8] and identify key changes in viscoelastic properties that occur secondary to pathologies including multiple sclerosis, Parkinson’s disease, and neoplasms [9,10,11]. The first application of MRE to the orbit utilized an ex vivo bovine globe to demonstrate the technical feasibility of quantifying viscoelastic parameters of the eye [12], suggesting potential application in the imaging of human orbits. However, there is a paucity of literature regarding the in vivo implementation of MRE for the human orbit and periorbital tissues, despite its potential to provide valuable insights into the biomechanical properties of these tissues in both healthy and diseased states. The structure and composition of orbital and ocular tissues are altered in many pathologic conditions, particularly various types of fibrosis and gliosis, including thyroid eye disease [13,14]. Previous studies have utilized fast cinematic 3D T1-weighted MR images to acquire volumetric information about and subsequently quantify soft tissue deformation during directional gaze [15]. While this method was shown to be effective in measuring three-dimensional motion of orbital soft tissues, subject cooperativity and variations in individual movement limit its generalizability. To address these challenges, it is important that appropriate equipment be developed for MRE of the orbit. Normal and pathologic viscoelastic parameters of ocular and orbital tissues must also be defined to aid in diagnosis; thus, it is crucial to develop a standardized technique for accurately determining the viscoelastic properties of orbital tissues. In this study, we describe the technical details as well as preliminary findings of shear wave MRE of the orbits in two healthy individuals. This preliminary attempt at performing MRE of the orbital region yielded stiffness maps of the globe but was technically limited in characterizing the adnexal soft tissues of the orbit. With further improvements, the quantification of viscoelastic properties of orbital tissues using MRE holds potential to significantly enhance diagnostic accuracy for conditions such as thyroid eye disease and orbital tumors secondary to the ability of MRE to detect subtle changes in tissue stiffness. The existing bibliography of MRE research supports the tailoring of equipment technical parameters to the organ or tissue being imaged [4,5,6,7]. Such optimization of image sequences, driver type and placement, and stiffness thresholds is especially important for orbit MRE due to the small and delicate nature of the orbital tissues. While a commercially available liver driver was used to demonstrate the feasibility of orbital MRE, the development of dedicated devices could be warranted based on the preliminary results shown in this study. The implementation of such devices may result in higher image quality and increased sensitivity in detecting pathologies affecting the viscoelastic properties of orbital tissues. Despite the promising initial results, there are several limitations to this study. The primary limitation of our study is a small sample size consisting of two healthy volunteers, which limits the generalizability of our findings. Additionally, this study was not focused on optimizing sequences, driver positions, or other technical parameters. Further research should initially focus on identifying effective technical parameters and elastography technique. This may lead to higher-resolution imaging and improved motion compensation techniques, thereby enhancing the utility of MRE in orbital imaging. Once the technical parameters are adequately defined, the optimized technique can be utilized to evaluate the utility of orbit MRE in the context of various orbital and ocular pathologies, such as thyroid eye disease, orbital tumors, and other inflammatory or neoplastic conditions. Similar to previous studies utilizing MRE in imaging of the head and neck, driver positioning and subject cooperation pose potential issues to widespread implementation of this technology. In conclusion, our findings provide evidence supporting the feasibility of orbital MRE in characterizing the viscoelastic properties of ocular tissues. By building on these results, addressing the above limitations, and extending the scope of the research to patients with pathological conditions, orbit MRE could be positioned as a valuable tool in both diagnostic and research-related settings. Specifically, this technique holds promise for studying biomechanical alterations associated with conditions such as glaucoma, diabetic retinopathy, and thyroid eye disease, where changes in tissue stiffness may serve as early biomarkers of disease progression. By enabling noninvasive assessment of these properties, orbital MRE could facilitate earlier diagnosis, improve disease monitoring, and contribute to the development of novel therapeutic strategies.

## Data Availability

Not applicable.
